# Human IgE does not bind to human FcRn

**DOI:** 10.1038/s41598-021-03852-1

**Published:** 2022-01-07

**Authors:** Maximilian Brinkhaus, Elvera J. van der Kooi, Arthur E. H. Bentlage, Pleuni Ooijevaar-de Heer, Ninotska I. L. Derksen, Theo Rispens, Gestur Vidarsson

**Affiliations:** 1 Department of Experimental Immunohematology, Sanquin Research and Landsteiner Laboratory, Amsterdam UMC, University of Amsterdam, Plesmanlaan 125, 1066 CX Amsterdam, The Netherlands; 2 Department of Immunopathology, Sanquin Research and Landsteiner Laboratory, Amsterdam UMC, University of Amsterdam, 1066 CX Amsterdam, The Netherlands

**Keywords:** Biochemistry, Blood proteins, Immunology

## Abstract

The neonatal Fc receptor (FcRn) is known to mediate placental transfer of IgG from mother to unborn. IgE is widely known for triggering immune responses to environmental antigens. Recent evidence suggests FcRn-mediated transplacental passage of IgE during pregnancy. However, direct interaction of FcRn and IgE was not investigated. Here, we compared binding of human IgE and IgG variants to recombinant soluble human FcRn with β2-microglobulin (sFcRn) in surface plasmon resonance (SPR) at pH 7.4 and pH 6.0. No interaction was found between human IgE and human sFcRn. These results imply that FcRn can only transport IgE indirectly, and thereby possibly transfer allergenic sensitivity from mother to fetus.

## Introduction

The neonatal Fc receptor (FcRn) is a mostly intracellularly expressed^1–3^, membrane-associated receptor, which is best known for mediating the extraordinarily long half-life of IgG^4–6^ and placental transport of thereof^3,7–9^. IgG is one of the most abundant serum proteins and the most abundant immunoglobulin found in human serum^10^. Mutational analysis have led to the identification of IgG1-Fc variants influencing IgG binding to FcRn. IgG1-MST-HN has been found to exhibit increased binding to FcRn^11^, whereas IgG1-IHH cannot bind FcRn^12^.

IgG can mediate a non-cellular response by engaging the complement system as well as it can bind Fc gamma receptors (FcγRs) and cross-link them on effector cells in the form of IgG immune complexes (ICs), triggering cellular effector functions^10^. In contrast to IgG, IgE is the immunoglobulin with the lowest abundance in serum. Next to its role in the defense of parasites^13^, IgE is known for its involvement in immune reactions against environmental antigens, causing type I hypersensitivity^14,15^. There are two main IgE Fc receptors, the high affinity FcεRI and the low affinity FcεRII (CD23). Cross-linking upon binding of antigens to FcεRI-bound IgE or binding of IgE ICs on the surface of e.g. basophils and mast cells has been reported to initiate cellular immune responses^14^. CD23 on the other hand exists in both in a trimeric membrane-bound and soluble mono- and trimeric forms, regulating IgE synthesis and homeostasis^16^. Membrane-bound CD23 is expressed on B cells and intestinal epithelial cells, where it also controls IgE synthesis and mediates transfer of IgE-ICs to the intestinal lumen, respectively^14,17^.

Whereas it is clear that the active transport of IgG across the placenta to the unborn is FcRn-mediated^8,9,18^, transport of other isotypes such as IgA and IgM is generally not considered relevant, most likely passive, as only a small fraction of what is found in maternal sera can be found in cord blood^19^. However, FcRn involvement has been reported for the transfer of tolerance to food allergens from mother to offspring in mice^20^ as well as for the transfer of IgE in anti-IgE IgG/IgE ICs in mice^21^ and humans^22^. A recent study suggested FcRn-dependent placental transport of IgE from mother to offspring in mice^23^.

In this study we investigated the binding of human IgE and IgG variants to human sFcRn using SPR aiming to complement the already published cellular data from a physicochemical point of view.

## Materials and methods

### Generation of anti-biotin IgG1-Fc variants, anti-biotin IgE and human sFcRn

Linear DNA strands encoding for mutated IGHG1*03 and IGHE*02 Fc-regions were ordered from Integrated DNA Technologies and cloned into a pcDNA3.1 expression vector containing anti-biotin heavy chain variable regions obtained from^24,25^, as described previously^26,27^. Linear DNA strands encoding for the soluble FcRn α-chain with a C-terminal BirA deca-Histidine (His) tag and ß2-microglobulin were ordered accordingly and cloned separately into pcDNA3.1 expression vectors, as described elsewhere^28^. In brief, expression vectors and DNA inserts were digested with EcoRI and NheI FastDigest restriction enzymes (Thermo Scientific). The expression vector backbone was isolated by gel purification using a 1% UltraPure agarose (Thermo Scientific) gel with 1:10.000 SYBR Safe (Invitrogen). DNA was extracted from the gel using the NucleoSpin Gel and PCR clean-up kit (Macherey–Nagel) according to the manufacturer’s protocol. The DNA fragments were isolated using the same kit but without prior gel purification.

The DNA fragments were ligated into the pcDNA3.1 backbone overnight at 16 °C using T4 DNA Ligase (New England Biolabs) in 1 × T4 DNA Ligation buffer (New England Biolabs) with a molar ratio of insert to vector of 3:1. 5 µL of the ligation reaction was transformed into 50 µL DH5α competent cells (Thermo Scientific) by heat shock.

The cells were plated on LB-agar plates containing 50 µg/mL ampicillin (Thermo Scientific) and incubated overnight at 37 °C. Colonies were picked and grown in 2 mL LB medium containing 50 µg/mL ampicillin (Thermo Scientific) overnight at 37 °C, shaking at 180 RPM. DNA was isolated from the bacterial culture using the NucleoSpin Plasmid EasyPure kit (Macherey–Nagel) according to the manufacturer’s protocol and sequenced.

Sequence-confirmed DNA was used for transformation of DH5α competent cells as described above and colonies were picked and grown in a 5 mL preculture. This was subsequently used to inoculate 200 mL LB medium containing 50 µg/mL ampicillin (Thermo Scientific), after which the culture was grown overnight at 37 °C, shaking at 180 RPM. DNA was isolated using the NucleoBond Xtra Maxi kit (Macherey–Nagel) according to the manufacturer’s protocol and again sequenced.

### Production of anti-biotin IgG1-Fc variants, anti-biotin IgE and BirA-His-tagged human sFcRn

Antibodies were produced as described previously^26^. In brief, 31.35 µg of the heavy chain vector, 37.65 µg light chain vector (pcDNA3.1 anti-biotin VLCL^24,25^) and 31 µg pSVLT/p21/p27 mix^29^ were added to 6.66 mL opti-MEM (Thermo Scientific) per 100 mL of transfection cell culture. 300 µL Polyethylenimine (PEI) MAX (linear, MW 4.000, Polysciences) was added, the mixture was immediately vortexed and incubated for 20 min at room temperature. 100 mL of HEK293F cells (Thermo Scientific) at 1*10^6^ cells/mL in fresh FreeStyle 293 Expression Medium (Thermo Fisher Scientific) were transfected with the mixture and incubated at 37 °C at 8% CO2 and shaking. After 4 h, 100 units/mL penicillin and 100 µg/mL streptomycin (Thermo Fisher Scientific) were added to the culture. Human sFcRn was produced as described previously^28^, using equimolar amounts of both the soluble FcRn α-chain and ß2-microglobulin expression vectors. The culture supernatants were harvested 6 days after transfection by spinning down the cells twice for 5 min at 3.100 × g and filtering through a 0.45 µm syringe filter (Whatman).

### Purification of recombinant anti-biotin IgGs and human sFcRn

Anti-biotin IgGs were purified from culture medium with AKTA prime (GE Healthcare) by affinity chromatography using either a 5 mL HiTrap HP protein A (IgG1-WT and -MST-HN) or protein G (IgG1-IHH) column (GE Healthcare), as described previously^30^, or a HisTrap HP column (sFcRn) (GE Healthcare). Fractions containing the antibodies or sFcRn were combined and concentrated using a 10 K MWCO Pierce Protein Concentrator PES (Thermo Scientific). Antibodies were fractionated by HPLC-SEC using an AKTA UPC-900, P-920 and Frac-950 (GE Healthcare) with a Superdex 200 10/300 GL column (GE Healthcare). Monomeric fractions were combined, antibodies were dialyzed to 5 mM sodium acetate (pH 4.5) and human sFcRn to 1xPBS (Fresenius Kabi) using either a 10 K MWCO Slide-A-Lyzer dialysis cassette (Thermo Scientific) overnight at 4 °C or using a 7 K MWCO Zeba Spin desalting column (Thermo Scientific) according to the manufacturer’s protocol. Protein concentrations were measured using a Nanodrop 2000c spectrophotometer (Thermo Scientific), adjusted to 1 mg/mL and aliquoted to 20 µL working stocks. Working stocks were stored at − 20 °C until assayed.

### HPLC-SEC

Analytical HPLC-SEC runs were performed using an Agilent 1260 Infinity II HPLC system (Agilent) coupled to a SDP-20A UV/Vis detector (SHIMADZU), a miniDAWN (Wyatt Technologies) and an Optilab (Wyatt Technologies). 16.67 µg of each molecule were assayed on a Superdex 200 10/300 GL (Cytiva) in 1xPBS (Fresenius Kabi) at a flow speed of 0.75 mL/min.

### SDS-PAGE

Each antibody was tested in SDS-PAGE under reducing and non-reducing conditions. Samples were incubated for 5 min at 70 °C or 95 °C in the presence of 20 mM Iodoacetamide (to prevent reduction during denaturation)^31^ or 0.25% (w/v) ß-Mercaptoethanol in NuPAGE™ LDS Sample Buffer (Thermo Scientific) for non-reduced and reduced conditions, respectively. Samples were loaded on a NuPAGE™ 4–12% Bis–Tris Gel (Invitrogen) and run with MOPS SDS running buffer (Thermo Scientific) for 10 min at 100 V followed by 45 min at 120 V. The gel was stained overnight in Blue-Silver solution (10% phosphoric acid, 10% ammonium sulfate, 0.12% CoomassieBlue G-250 (Sigma-Aldrich) and 20% methanol (Thermo Scientific) and thoroughly destained in distilled water.

### Native PAGE and Western Blot

IgE-containing serum sample^32^ and IgE-containing culture supernatant were diluted in 2 × Native Tris–Glycine Sample Buffer (Life Technologies) and run for 90 min at 150 V on a NuPAGE 3–8% Tris–Acetate Gel (Thermo Scientific). The gel was transferred to a iBlot2 NC Mini Stack (Thermo Scientific) and blotted using an IBlot2 Gel Transfer device (Thermo Scientific). The membrane was blocked for 30 min using 1 × Western Blocking Reagent (Roche), followed by incubation with 1:500 anti-hIgE (MH25-1) (Sanquin) in the same solution for 1 h. After extensive washing, the membrane was incubated with 1:500 polyclonal goat anti-mouse HRP (Dako) for 1 h in the same blocking solution. After another extensive washing step, the blot was developed using Pierce ECL Western Blotting Substrate (Thermo Scientific) according to manufacturer’s instructions.

### SPR

Affinity measurements to human sFcRn in SPR were performed using an IBIS MX96 (IBIS Technologies) device and a Continuous Flow Microspotter (Wasatch Microfluidics). BSA-biotin (ITK Technologies) was spotted at four concentrations each on a SensEye G Easy2Spot (SensEye) in 10 mM sodium acetate at pH 4.5 with 0.075% (v/v) Tween (80) in 2 × dilution series starting at 60 nM. Anti-biotin IgG or anti-biotin IgE was injected at a concentration of 100 nM or 2 × diluted culture supernatant, respectively. Titration of human sFcRn was performed by injecting seven concentrations of human sFcRn, from 15.63 nM to 1000 nM (2 × dilution series) in 1xPBS containing 0.075% (v/v) Tween (80) at pH 7.4 or pH 6.0. The sensor was regenerated between the cycles by two subsequent injections of 20 mM Tris–HCl, 150 mM NaCl pH 8.8 and 20 mM H_3_PO_4_ pH 2.4. K_D_ values were calculated by fitting a Langmuir 1:1 binding model to a R_max_ = 700, as described previously^33^, using Scrubber software version 2 (BioLogic Software) and excel. IgE culture supernatant was confirmed to contain anti-biotin IgE by injection of 100 nM of anti-hIgE (MH25-1) (Sanquin) or soluble FcεRI α-chain (soluble FcεRI) (Sino Biologicals) in 1xPBS containing 0.075% (v/v) Tween (80) at pH 7.4 or pH 6.0 after antigen capture of antibodies.

## Results

### Anti-biotin IgG variants and IgE are functional and show expected molecular assembly

**Figure 1:**
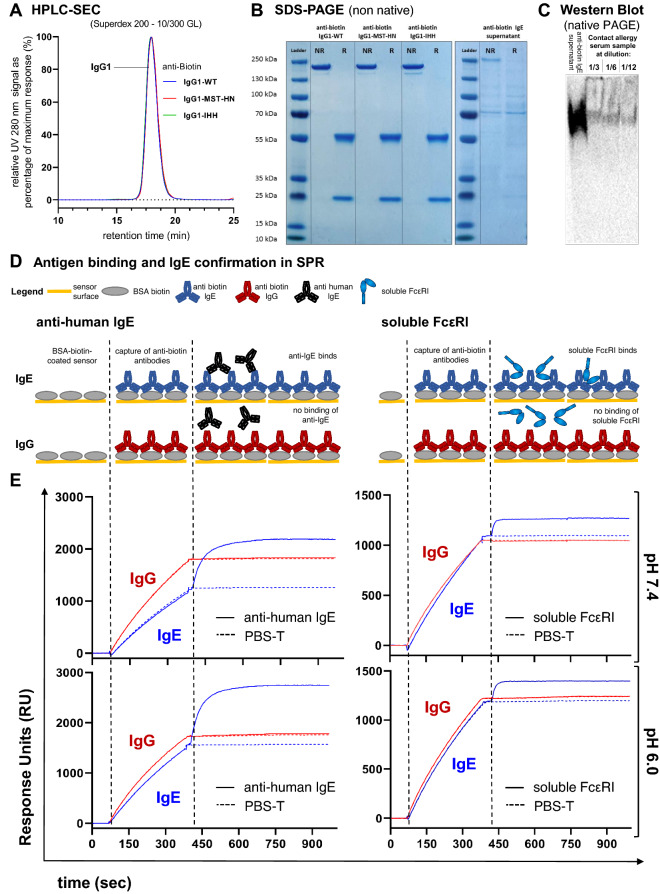
Confirmation of antibody integrity and antigen binding in SPR. (**A**) HPLC-SEC chromatograms of the IgG variants as relative UV280 nm signal normalized to maximum response. The chromatograms of molecules overlap. (**B**) SDS-PAGEs under non-reducing (*NR*) and reducing (*R*) conditions. Shown are two independently prepared SDS-PAGEs of purified V-gene matched IgG variants (left) and the IgE supernatant (right). The uncropped original images showing the relevant gel parts can be found in Supplementary Fig. 1A and B, respectively. (**C**) IgE-specific Western Blot after native PAGE of anti-biotin IgE supernatant next to contact allergy serum sample^32^. The uncropped original image showing the relevant lanes can be found in Supplementary Fig. 1C. (**D**) Schematic overview of SPR experiments showing anti-biotin antigen binding and presence of human IgE in culture supernatants. (**E**) Sensorgrams of one representative of three independent experiments showing binding of anti-IgE antibody (left) and soluble FcεRI (right) at pH 7.4 and pH 6.0 after capturing anti-biotin IgE from culture supernatant in comparison to IgG at 100 nM.

Anti-biotin IgG1 variants were tested in HPLC-SEC in order to confirm their integrity. All molecules showed expected sizes and were monomeric (Fig. 1A). Furthermore, integrity and size of all IgG molecules assayed were confirmed in SDS-PAGE (Fig. 1B, left). For the IgE supernatant we observed some additional bands on the gel (Fig. 1B, right), presumably due to other protein species in the culture supernatant and the fact that a non-native gel was used. We therefore further confirmed the integrity of the IgE in the culture supernatant in an IgE-specific Western Blot after separation on a native gel next to a contact allergy serum sample (Fig. 1C) of a previously described patient cohort^32^. We then employed our SPR platform to test antigen binding and further validate the molecular identity of the IgE in the culture supernatant. As shown in the schematic overview in Fig. 1D, BSA-biotin was spotted on the sensor and purified anti-biotin IgG or IgE culture supernatant was injected, followed by a subsequent injection of anti-hIgE or PBS (left panel) or—in independent experiments—soluble FcεRI or PBS (right panel). Both anti-biotin IgG and IgE bound BSA-biotin at pH 7.4 and pH 6.0. Only the IgE was recognized by anti-IgE antibody and soluble FcεRI, confirming its molecular identity (Fig. 1E).

### Anti-biotin IgE does not bind to human sFcRn

**Figure 2:**
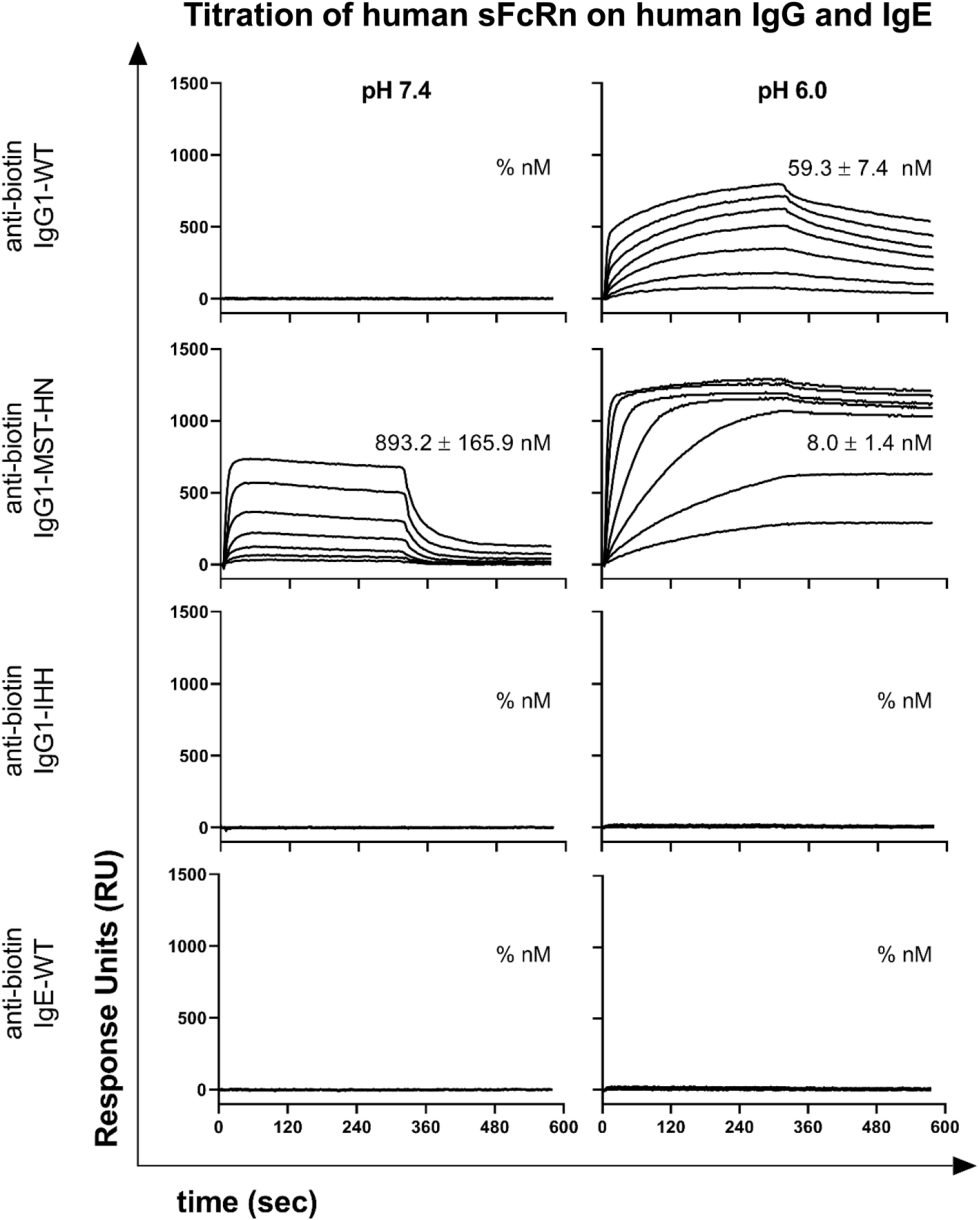
Titration of human sFcRn in SPR reveals no binding to IgE at pH 7.4 or 6.0. Anti-biotin IgG variants and IgE were captured on a sensor with different concentrations of BSA-biotin spotted, leading to comparable levels of antigen-bound IgG and IgE (not shown due to blank subtraction). Human sFcRn was injected in a twofold serial dilution covering a concentration range from 15.63 to 1000 nM and K_D_ values were calculated fitting a 1:1 Langmuir binding model after blank subtraction. A representative of three independent experiments is shown.

Next, we tested binding of the antibodies to human sFcRn in our SPR system after antigen capture, as described previously^34^. In order to have a valid set of controls, we included two previously described IgG1-Fc variants, anti-biotin IgG1-MST-HN^11^ and IgG1-IHH^12^, which have enhanced^11^ and no binding^12^ to FcRn. We captured the antibodies on the sensor in the same manner as in Fig. 1E, reaching immobilization levels allowing to test binding of sFcRn. IgG1-MST-HN bound to human sFcRn at pH 7.4, whereas none of the other antibodies tested exhibited binding at physiological pH. At pH 6.0, strong binding was observed to anti-biotin IgG1-MST-HN and IgG1-WT, whereas human sFcRn did not bind IgG1-IHH. The observed binding profiles and K_D_ values of the IgG variants are in line with earlier published results^11,12,35^. Importantly, no binding of human sFcRn was observed to IgE (Fig. 2).

## Discussion

Human FcRn mediates placental transcytosis of IgG from mother to unborn^3,7–9^. Yet, it remains not fully understood how and to what extent isotypes other than IgG, e.g. IgE, cross the placental barrier^22,23,36^. In this study, we compared binding of human sFcRn to different Fc variants of IgG1 and to IgE at both neutral and acidic pH. Unlike IgG, which shows a clear pH-dependent binding to FcRn, no binding was seen to human IgE.

The concept of FcRn-dependent transfer of tolerance from mother to offspring is well established in mice: After sensitization of mothers, antigen-specific IgG has been described to confer tolerance to e.g. ovalbumin (OVA) or food allergens to offspring. Transfer of such protective IgG and IgG-ICs has been reported to occur via placental passage and via the mother milk, respectively, both in a FcRn-dependent manner^20,37–39^.

The presence of IgE in cord blood has been reported in humans, albeit at low concentrations compared to paired maternal blood^36,40–42^. Possibly, some placental transcytosis of IgE molecules takes place^22,23,36,40^. Evidence has been reported that this happens in a FcRn-dependent manner in humans^22^ and in mice^21,23^. Two recent studies implied a role of maternal IgE in priming allergic responses in offspring, providing evidence for such indirect FcRn-dependent placental transport in mice^21,23^ and suggested a direct FcRn-mediated transport of IgE^43^. It is unclear to which extent the high amounts of antigen-specific IgE administered to the parental animals during pregnancy in these studies^21,23^ – more than 100-fold higher than naturally occurring total IgE levels in mice^44^ – can be extrapolated to more physiological concentrations, as recently noted elsewhere^45^.

If direct FcRn-mediated transport of IgE does not occur, are there other ways FcRn might play a role in IgE transport across the placenta? One possibility is FcRn-mediated placental passage of complexes of IgE with IgG, forming anti-IgE IgG/IgE complexes^21,22^. The presence of such complexes in serum is controversial^46^. Next to anti-IgE IgG/IgE ICs, FcRn-mediated placental passage of antigen-specific IgE could also occur in the form small ICs formed by IgG and IgE binding the same antigen. Interestingly, a study by Weil et al. provides indirect evidence for this scenario in the context of parasitic infection. Although they found remarkable levels of parasitic IgE in cord blood, often exclusively antigen-specific but generally ~ 50–100 times less than found in maternal blood, the data was interpreted as an antigen-specific IgE response originating from the fetus. However, such IgE levels could also be interpreted as evidence for ICs formed by IgG and IgE bound to the same antigen^36^, but do not favor the hypothesis of IgE being commonly transported across the placental barrier.

Invariably, the relative total IgE concentrations found in cord blood compared to matched parental samples are very low^36,40^, comparable to or even lower than relative levels of IgA or IgM, respectively^19^, not suggestive of an active, directly FcRn-mediated placental transport as described for IgG^3,7–9,19^.

Our results complement previously published data showing no binding of hIgE to hFcRn when overexpressed on MDCK cells by FACS^22^ with binding data to human sFcRn in SPR at both pH 7.4 and pH 6.0. We conclude that it is very unlikely that human FcRn directly mediates placental transcytosis of human IgE, but rather through ICs containing IgG, possibly as anti-IgE IgG/IgE ICs, as suggested elsewhere^21,47^.

## Data availability 

The datasets generated during and/or analyzed during the current study are available from the corresponding author on reasonable request.

## Supplementary Information

Supplementary Information.

## Abbreviations

BSA: Bovine serum albumin
DNA: Deoxyribonucleic acid
Fc: Fragment crystallizable
FcεR: Fc epsilon receptor
FcγR: Fc gamma receptor
FcRn: Neonatal Fc receptor
FCS: Fetal calf serum
h: Hour
HEK: Human embryonic kidney
His: Histidine
HPLC: High performance liquid chromatography
IC: Immune complex
Ig: Immunoglobulin
K_D_: Dissociation constant
min: Minute
MWCO: Molecular weight cut-off
nM: Nanomolar
OVA: Ovalbumin
PAGE: Polyacrylamide gel electrophoresis
PEI: Polyethylenimine
PBS: Phosphate buffered saline
PCR: Polymerase chain reaction
RPM: Rounds per minute
RT: Room temperature
SDS: Sodium dodecyl sulfate
sec: Second
SEC: Size exclusion chromatography
SPR: Surface plasmon resonance
V: Volt
WT: Wild type
