# Supplementary material for: Human IgE does not bind to human FcRn
Source: Sci Rep. 2022 Jan 7;12:62. doi: 10.1038/s41598-021-03852-1 (PMC8741920; doi:10.1038/s41598-021-03852-1)
Supplement: Supplementary file 1 — Supplementary Information. [file 41598_2021_3852_MOESM1_ESM.pdf]

Supplementary Figure 1

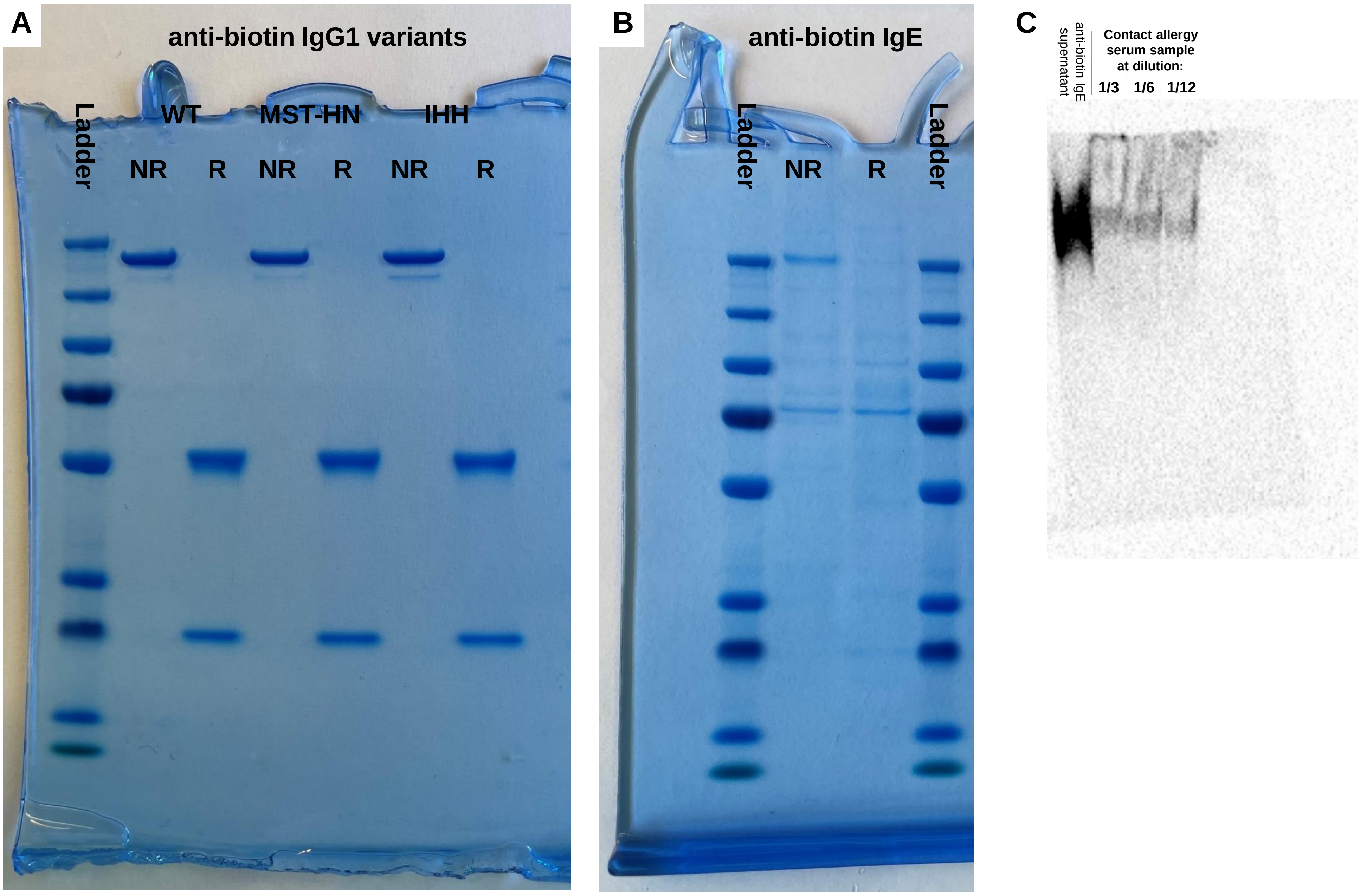

Supplementary Figure 1. Uncropped images of SDS-PAGEs from Fig. 1B and Western Blot from Fig. 1C. SDS-PAGEs under non-reducing (NR) and reducing (R) conditions. Shown are two independently prepared SDS-PAGEs of (A) purified V-gene matched IgG variants and (B) the IgE supernatant. (C) IgE-specific Western Blot after native PAGE of anti-biotin IgE supernatant next to contact allergy serum sample.
